# Stabilizing Lithium–Sulfur Batteries in Carbonate Electrolytes via DODT–PEG Interphase Engineering and Ultramicroporous Carbon Confinement

**DOI:** 10.1021/acsomega.6c06179

**Published:** 2026-08-25

**Authors:** Derek Ovc-Okene, Lakshmi Shiva Shankar, Péter Nagy, Marton Szabados, Dávid Ugi, Ákos Szabó, Béla Iván, Robert Kun

**Affiliations:** † Renewable Energy Research Group, Institute of Materials and Environmental Chemistry, HUN-REN Research Centre for Natural Sciences, Magyar tudósok krt. 2, Budapest H-1117, Hungary; ‡ Department of Chemical and Environmental Process Engineering, Faculty of Chemical Technology and Biotechnology, Budapest University of Technology and Economics, Műegyetem rkp. 3, Budapest H-1111, Hungary; § Zalaegerszeg Innovation Park, Széchenyi István University, Dr. Michelberger Pál út 3, Zalaegerszeg H-8900, Hungary; ∥ Department of Physical Chemistry and Materials Science, University of Szeged, Rerrich Béla tér 1, Szeged H-6720, Hungary; ⊥ Department of Applied and Environmental Chemistry, University of Szeged, Rerrich Béla tér 1, Szeged H-6720, Hungary; # Polymer Chemistry and Physics Research Group, Institute of Materials and Environmental Chemistry, HUN-REN Research Centre for Natural Sciences, Magyar tudósok Krt. 2, Budapest H-1117, Hungary; ∇ Department of Materials Physics, Eötvös Loránd University, Pázmány Péter sétany 1/a., Budapest 1117, Hungary; ○ Sustainability Competence Centre, Széchenyi István University, Egyetem square 1, Győr H-9026, Hungary

## Abstract

Stabilizing lithium–sulfur batteries in carbonate-based electrolytes requires simultaneous control over sulfur behavior and interfacial chemistry. In this work, we present a cooperative strategy that combines a poly­(vinylidene fluoride) (PVDF)-derived ultramicroporous carbon (UMC) sulfur host with a 3,6-dioxa-1,8-octane-dithiol -poly­(ethylene glycol) (DODT–PEG) copolymer electrolyte additive to address these challenges in a unified manner. The ultramicroporous structure of UMC physically confines sulfur species within subnanometer pores, limiting polysulfide formation and outward diffusion, while the DODT–PEG copolymer modifies the cathode/electrolyte interphase to promote ion-conductive pathways and stabilize sulfur redox reactions. Through structural, electrochemical, impedance, and postmortem analyses, the distinct roles of sulfur confinement and interphase regulation are identified and correlated with improved electrochemical behavior. The combined system enables high reversible sulfur utilization, stable cycling with a Coulombic efficiency of >99%, and enhanced rate capability. Li//Li symmetric cell analysis further reveals that the beneficial effect of the copolymer additive is localized at the cathode rather than at the lithium surface. This work demonstrates that coordinated host design and electrolyte engineering provide a simple and scalable route for improving Li–S battery performance in conventional carbonate electrolytes.

## Graphical abstract

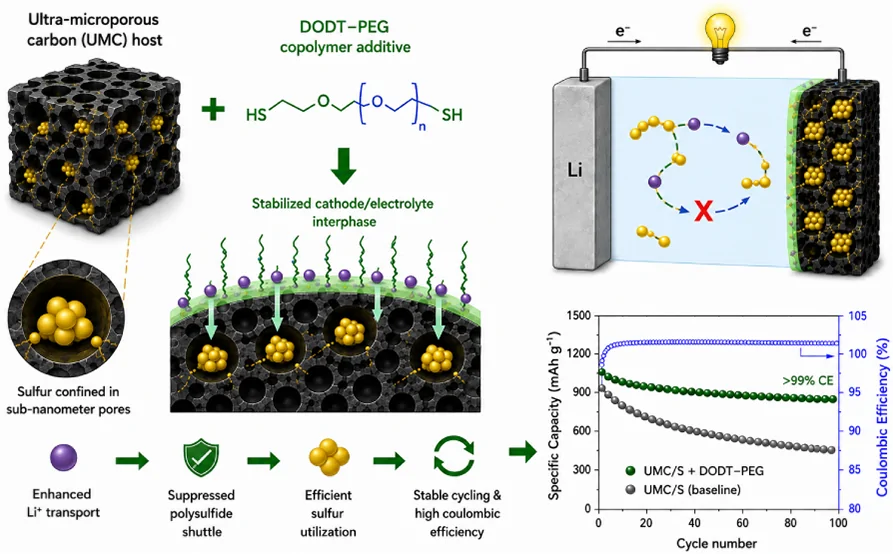

## 1 Introduction

Lithium–sulfur (Li–S) batteries are a focal point of current research, praised for their impressive energy density, abundant sulfur resources, cost-effectiveness, and eco-friendliness. Many experts predict that Li–S batteries could 1 day replace Li-ion technology, which has already had a transformative impact on electronics and modern life.^1–5^ Although sulfur offers a theoretical specific capacity, which is 8–10 times greater than current commercial cathodes,^6^ several intrinsic challenges limit the practical use of Li–S batteries.

First, the poor conductivity of sulfur (S_8_) and lithium sulfide (Li_2_S) leads to significant kinetic challenges in the complex conversion reactions of sulfides, resulting in poor electrochemical performance, including low-capacity utilization and low practical energy density.^7,8^ Second, the high solubility of long-chain lithium polysulfides (LiPSs) in organic liquid electrolytes causes the shuttle effect, where soluble sulfur species migrate between the cathode and anode, degrading the reaction interface over repeated cycles.^9^ Finally, the substantial volume expansion (80%) during lithiation leads to a deterioration in the mechanical stability of sulfur cathodes.^10^

Consequently, most strategies for improving Li–S batteries have focused on addressing these challenges individually through the development of innovative composite cathodes,^11,12^ electrolyte additives,^13–16^ modified functional separators,^17–19^ and the stabilization of the lithium metal anode.^20–23^ However, in practical cells these issues occur simultaneously, and therefore solutions that address only one limitation often fail to provide long-term stability. Porous carbons have attracted considerable interest among the potential materials, owing to their extensive surface area and substantial pore volume, which facilitate the storage of large quantities of sulfur within the pores after sulfur impregnation.^24,25^ Porous carbons obtained from poly­(vinylidene fluoride) (PVDF) precursors have recently surfaced as viable sulfur hosts, providing benefits including a facile synthesis process and the utilization of an inexpensive precursor.^26–28^ Microporous carbon derived from PVDF exhibits a high average pore volume of 0.41 cm^3^ g^–1^ and features a narrow average pore size distribution of 0.55 nm.^29^ Such ultramicroporous structures are especially beneficial because they can physically confine small sulfur species and reduce the driving force for polysulfide dissolution into the electrolyte.

At the same time, the design of electrolyte additives for Li–S batteries holds great potential for mitigating the shuttle effect and protecting the Li anode, although it remains relatively underexplored.^30,31^ LiNO_3_ is a widely used additive in ether-based electrolytes for Li–S batteries, known for passivating the Li surface by converting reduced sulfide species into oxidized sulfate compounds (Li_2_S_*x*_O_*y*_), thereby forming a stable SEI layer.^32^ However, this layer suffers from low Li-ion conductivity and increasing resistance as the nitrate is consumed as the cell cycles. Other additives, such as lithium iodide, phosphorus pentasulfide, and tin iodide, have also demonstrated the ability to form protective layers on the Li anode. Nevertheless, the stability and ionic conductivity of these SEI layers, along with the cell’s cyclability and overall efficiency, require further optimization.^33,34^

Improvement and optimization of the electrolyte, in comparison to adjusting the solvents and salts in the electrolyte or creating new electrolytes, adding additives is a more inexpensive, practical, and effective technique to boost battery performance, along with the electrode materials and separators. The electrolyte not only transports Li^+^ but also has a high solubility for polysulfides, which promotes the electrochemical reaction process of the S cathode.^35^ Furthermore, the electrolyte has a significant influence on the battery’s safety, cycle stability, and high-temperature performance.^36^ Because of their functional groups, electrolyte additives might supply functional additive moieties, produce stable interfacial layers, and decrease the shuttle effect of LiPSs. Through covalently attaching sulfur, the insertion of organic functional groups is a possible approach for the direct chemical modification of LiPSs dissolved in electrolyte.^37,38^

Motivated by these considerations, this work presents a combined strategy that addresses both sulfur confinement and interfacial ionic transport simultaneously. A PVDF-derived ultramicroporous carbon (UMC) is used as a sulfur host to physically confine sulfur species within subnanometer pores, while a DODT–PEG copolymer additive (DcP) is introduced into the electrolyte to enhance Li^+^ transport and stabilize the cathode/electrolyte interphase. Both DODT and PEG were selected on the basis of our previous studies.^39,40^

The purpose of this study is therefore not to introduce a new carbon host or a new additive independently, but to demonstrate how the combination of host design and electrolyte design can combine to improve Li–S battery performance. We show, through structural, electrochemical, and postmortem analyses, that the combined 3S2UMC + DcP system provides improved reversible capacity, rate capability, and cycling stability by limiting polysulfide formation, enhancing interfacial Li^+^ transport, and preserving electrode integrity during cycling.

## 2 Experimental Methods

### 2.1 Materials

Sulfur (precipitated powder, reagent grade, Reanal, Hungary), 1 M LiPF_6_ in EC/DEC = 50/50 (v/v), battery grade (99%, Sigma-Aldrich), *N*-methyl-2-pyrrolidone (99.9%, Sigma-Aldrich), lithium foil (Sigma-Aldrich), carbon black (Super C65, Imerys), and poly­(vinylidene fluoride) (PVDF, 99.9%, Solvay) were used as received.

### 2.2 Preparation of the UMC and S/UMC Composite

UMC was synthesized from PVDF through a simple pyrolysis process. PVDF powder was directly carbonized at 800 °C for 2 h in a tube furnace under a flowing nitrogen atmosphere with a heating rate of 10 °C min^–1^. The resulting UMC, rich in ultramicropores, was obtained without any further treatment.

The prepared UMC was then used to incorporate small sulfur particles. A mixture of UMC and precipitated sulfur in a mass ratio of S:UMC = 3:2 was heated to 155 °C at 0.5 °C min^–1^ from room temperature for 20 h. The temperature then increased to 300 °C and maintained for 2 h to remove excess sulfur from the UMC surface. As a result, the S/UMC composite was obtained. The sample prepared at a sulfur-to-carbon mass ratio of 6:4 is referred to as *3S2UMC* throughout this manuscript. This synthesis route ensures that sulfur is incorporated primarily within the ultramicroporous structure of the carbon host.

### 2.3 Material Characterization

The porosity of the UMC, both before and after sulfur loading, was characterized by nitrogen adsorption/desorption measurements at −196.15 °C using an automatic volumetric instrument (NOVA 2000e, Quantachrome) after a 24-h degassing period at 110 °C. The specific surface area (S_BET) and pore size distribution were calculated using the Brunauer–Emmett–Teller (BET) method. The micropore volume (V_mic) was estimated using the Dubinin–Radushkevich (DR) model. The total pore volume (V_t) was determined from the amount of vapor adsorbed at p/p_0_ = 0.94, assuming the adsorbed gas occupies the pores as liquid.

Morphology and elemental mapping were examined using scanning electron microscopy (SEM, FEI Quanta 3D dual-beam) coupled with energy dispersive X-ray spectroscopy (EDAX, Oxford X-Max 20).

Thermal behavior was analyzed using thermogravimetric analysis (TA Instruments Discovery TGA) coupled with a mass spectrometer (Hiden Analytical, HPR-20 EGA). Measurements were conducted under argon containing 10% O_2_ (60 mL min^–1^) at heating rates of 5, 10, and 15 °C min^–1^.

Structural characterization was performed using X-ray diffraction (XRD) with Cu Kα radiation and Raman spectroscopy (Bruker Senterra II, 532 nm excitation, 10 mW laser power).

X-ray Photoelectron Spectroscopy (XPS) measurements were performed by using an Omicron EA 125 electron spectrometer in the “Fixed Analyser Transmission” mode; photoelectrons were excited by nonmonochromatized Mg Kα (1253.6 eV) radiation. The samples on aluminum foil supports were clamped to stainless steel sample plates. After measuring a survey spectrum, high resolution spectra were collected from the regions of interest (S 2p, C 1s, O 1s, F 1s, etc.). For recording the high-resolution spectra, the electron spectrometer was operated in the Constant Analyzer Energy mode with a “Pass energy” of 30 eV, providing resolution around 1 eV. The energy scale of the electron spectrometer was calibrated according to the ISO 15472 standard. These characterization techniques were used to verify sulfur confinement within the UMC pore structure and to monitor structural stability before electrochemical testing.

### 2.4 Electrode Formulation

The cathode slurry consisted of 80/10/10 wt % 3S2UMC, Super C65, and PVDF binder dissolved in *N*-methyl-2-pyrrolidone. After homogenization, the slurry was cast onto aluminum foil using the doctor-blade technique. The sulfur loading was approximately 1 mg sulfur cm^–2^. Although the present study was conducted at modest sulfur loading to isolate mechanistic effects, future work will examine whether the DcP-enabled cathode/electrolyte interphase remains effective at higher areal sulfur loading and under lean-electrolyte conditions more relevant to practical Li–S cells. The electrodes were dried at 55 °C for 24 h under vacuum and punched into disks with 16 mm diameter.

### 2.5 Coin Cell Assembly

CR2032 coin cells were assembled in an argon-filled glovebox (O_2_ and H_2_O < 0.1 ppm) using the prepared cathode, lithium metal anode, and Whatman glass fiber separator. Each cell contained 20 μL of electrolyte consisting of 1 M LiPF_6_ in EC/DEC (1:1 v/v), to ensure reliable ionic contact. For modified electrolyte experiments, 1% DcP additive was introduced into the electrolyte.

### 2.6 Electrochemical Measurements

Electrochemical impedance spectroscopy (EIS) was performed using a Bio-Logic VMP3 potentiostat over a frequency range of 1 MHz to 10 mHz with an excitation amplitude of 10 mV. Data were fitted using ZView software. Cyclic voltammetry (CV) measurements were conducted at a scan rate of 0.1 mV s^–1^ over a voltage window of 1.2–2.8 V vs Li/Li^+^. Galvanostatic charge/discharge cycling and rate capability tests were carried out using the same workstation at various current densities. These electrochemical methods were used to evaluate how sulfur confinement and the DcP additive influence reaction kinetics and interfacial behavior during cycling.

## 3 Results and Discussion

### 3.1 UMC Structure and Sulfur Confinement: Physical Evidence

The UMC host was synthesized from PVDF precursor via controlled carbonization, producing a dominant ultramicropore distribution (pore diameters <1 nm) and a high specific surface area (Figure 1a–b). Nitrogen adsorption/desorption isotherms and pore-size analysis (Figure 1a) demonstrate that the pore volume is concentrated in the ultramicroporous range, which is a key requirement for physically trapping small sulfur allotropes and limiting their dissolution into the electrolyte.^41–43^

**Figure 1:**
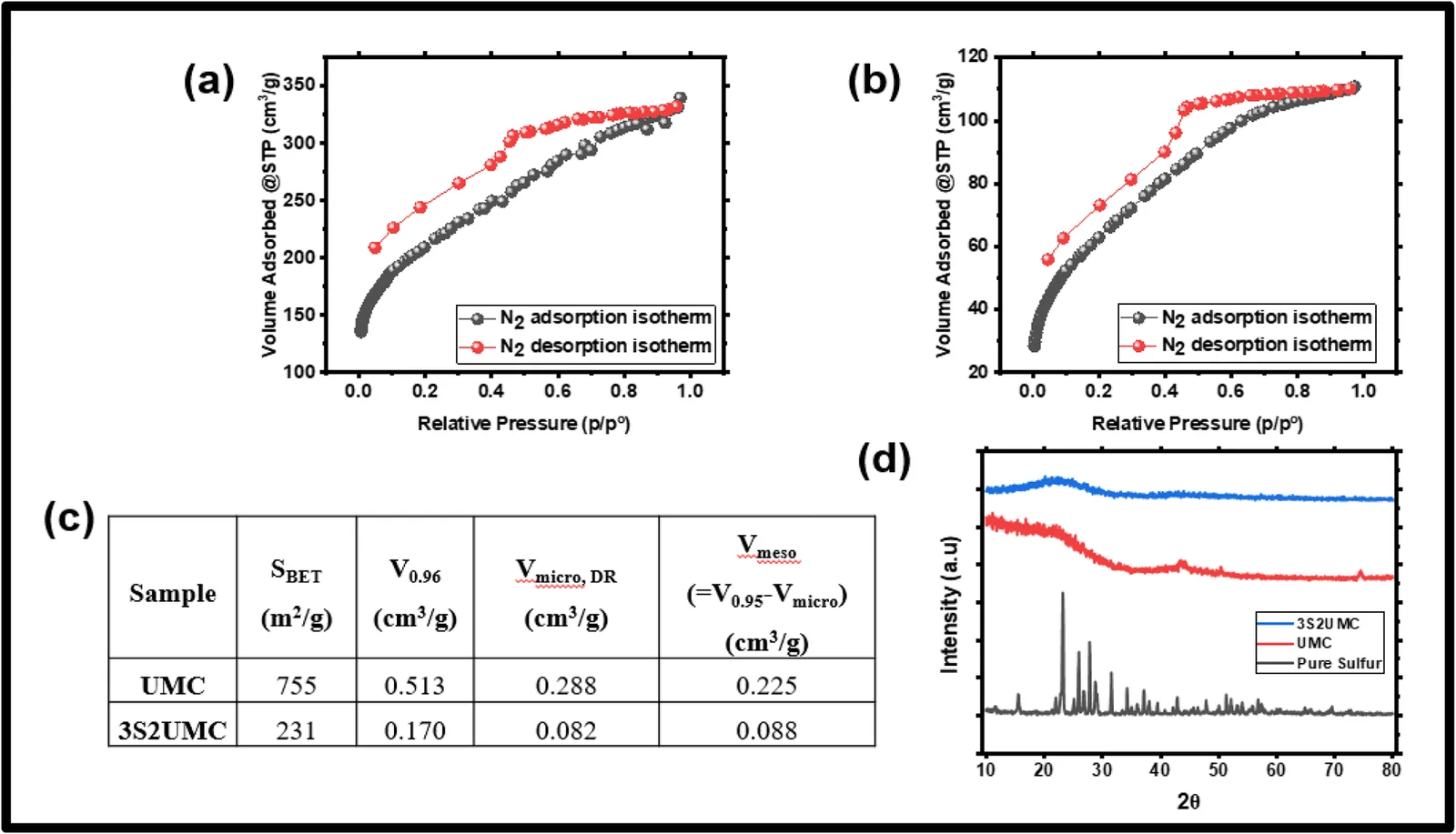
Pore architecture and structural proof. (a) Nitrogen adsorption–desorption isotherms for UMC exhibit a classic Type I curve. (b) Corresponding N_2_ isotherms for S/UMC composite. (c) Comparative BET parameters for UMC and sulfur-loaded 3S2UMC. (d) XRD showing broadened sulfur reflections consistent with highly dispersed sulfur.

After sulfur loading, the isotherms (Figure 1b) show a pronounced reduction in pore volume, indicating that sulfur has been incorporated into the pore network. The BET comparison (Figure 1c) confirms this pore-filling effect.

XRD analysis (Figure 1d) shows broadened sulfur reflections consistent with highly dispersed sulfur rather than large crystalline sulfur domains. This is important because highly dispersed sulfur generally increases sulfur utilization while decreasing the likelihood of large, poorly connected sulfur particles that lead to polarization and irreversible loss.

Thermogravimetric analysis provides complementary evidence (Figure 2). The sulfur loss temperature profile of 3S2UMC is shifted and suppressed relative to bulk sulfur, indicating that sulfur is not present mainly as surface-deposited crystalline particles but is strongly confined within the UMC pore structure. This confinement is directly relevant to Li–S cycling because it can slow the generation and outward diffusion of soluble long-chain polysulfides during discharge.

**Figure 2:**
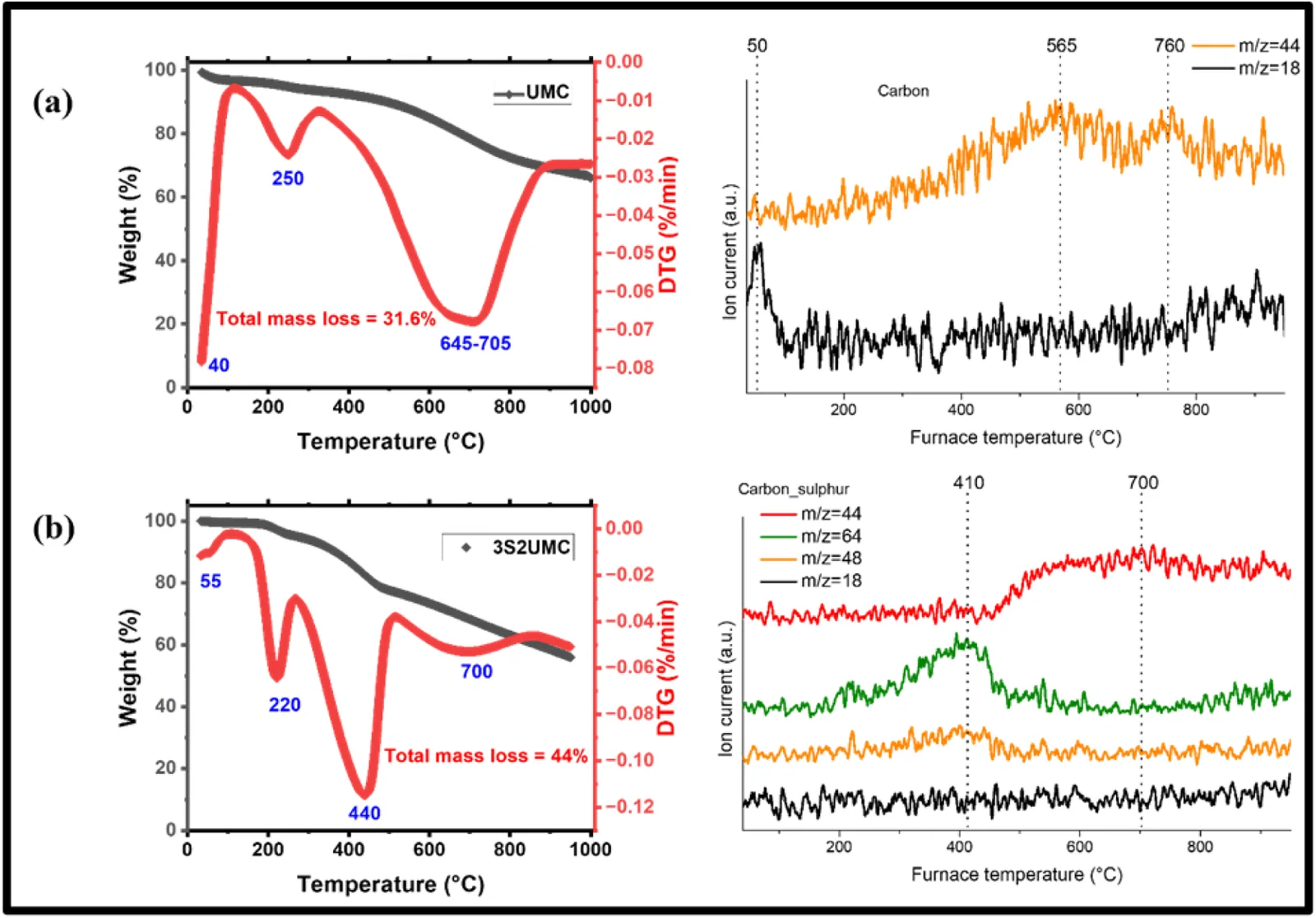
TG-DTG curves of (a) pristine UMC and (b) 3S2UMC.

The morphology and structure of the 3S2UMC composite were further analyzed using SEM. SEM images of pristine UMC (Figure 3a–c) and 3S2UMC (Figure 3d–f) reveal very similar surface morphologies, suggesting that sulfur incorporation does not alter the structural integrity of the carbon host. Elemental mapping (Figure 3g,h) confirms that sulfur, shown in purple in Figure 3h, is uniformly distributed throughout the carbon matrix, shown in red in Figure 3g, forming small, dispersed domains along the inner pore surfaces.

**Figure 3:**
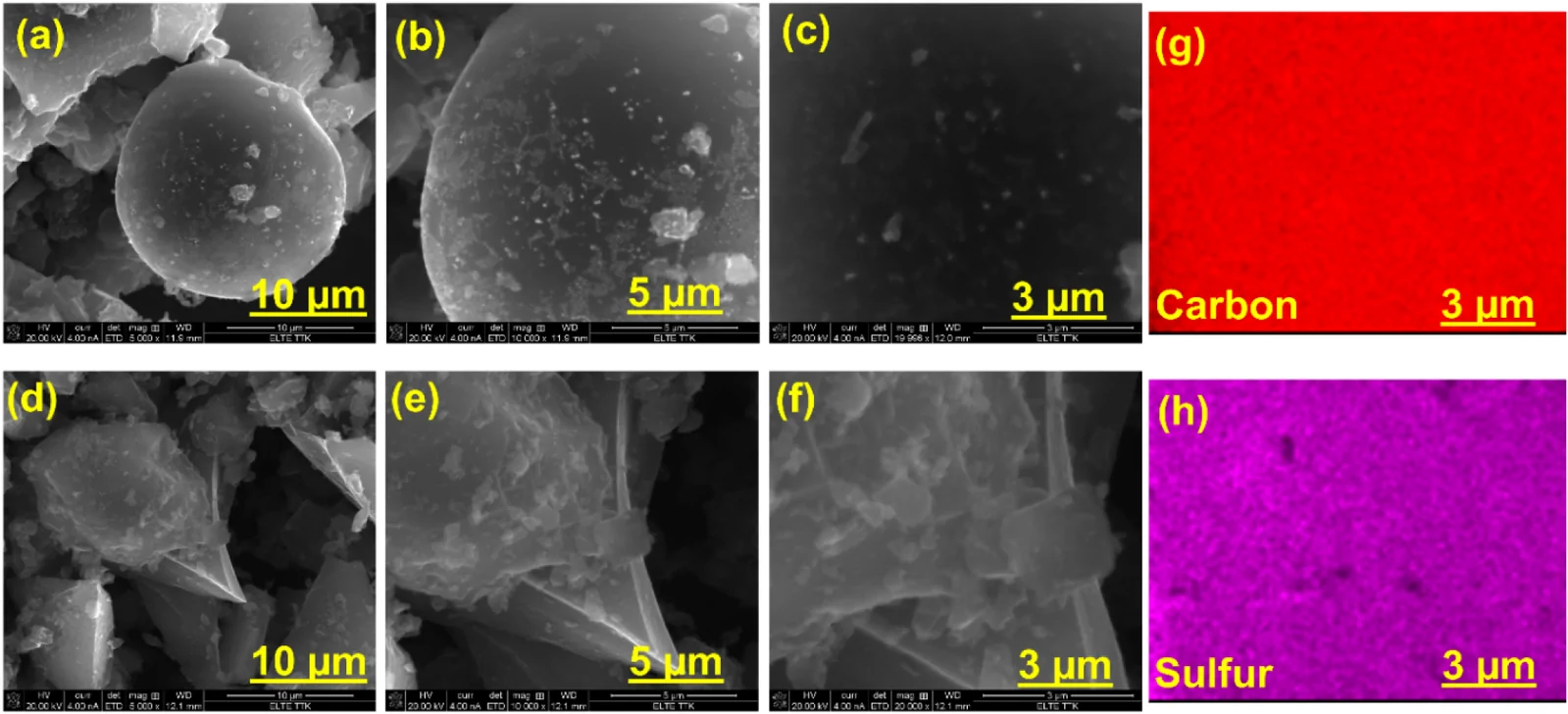
SEM images of (a–c) UMC, (d–f) 3S2UMC under different magnifications, and (g–h) elemental maps of carbon and sulfur in 3S2UMC.

Importantly, this homogeneous sulfur distribution remains even after heat treatment at 300 °C, which is far above the boiling point of sulfur. This observation indicates that sulfur is strongly retained within the pore network, likely due to the rough inner surface created during the pyrolytic synthesis of the UMC.

Taken together, the BET, XRD, TG, and SEM (Figures 1–3) results confirm homogeneous sulfur distribution within the UMC framework, indicating that the ultramicroporous carbon effectively confines sulfur and limits its dissolution. This structural confinement is expected to reduce the formation and escape of soluble polysulfides during cycling, which forms the basis for the electrochemical behavior discussed in the following sections. Additional SEM images and elemental analysis of the pristine 3S2UMC cathode are provided in Figures S2–S3, further confirming the homogeneous sulfur distribution within the UMC framework.

### 3.2 Baseline Electrochemical Behavior with and without DcP

With the structural confinement established, the next step is to test whether the DcP additive measurably changes the fundamental electrochemical behavior. Cyclic voltammetry (CV) was used as the first diagnostic because it directly reflects redox kinetics and polarization under identical material loading and cell design.

As shown in Figure 4a–b, the CV curves display the characteristic Li–S reduction and oxidation processes. Since the UMC-S shows only a single voltage plateau (below 2.0 V), it was hypothesized that the presence of S_4_ and S_2_ in UMC are the reason for the single discharge plateau, this is in line with the BET results. The DcP-containing cell shows clearer peak definition and reduced polarization compared to the standard electrolyte. This indicates that DcP does not merely change long-term stability; it affects the reaction pathway and/or interfacial kinetics already at the earliest stages of cycling. A simple way to interpret this is that DcP promotes a more favorable cathode/electrolyte interphase, reducing kinetic barriers for sulfur conversion reactions.

**Figure 4:**
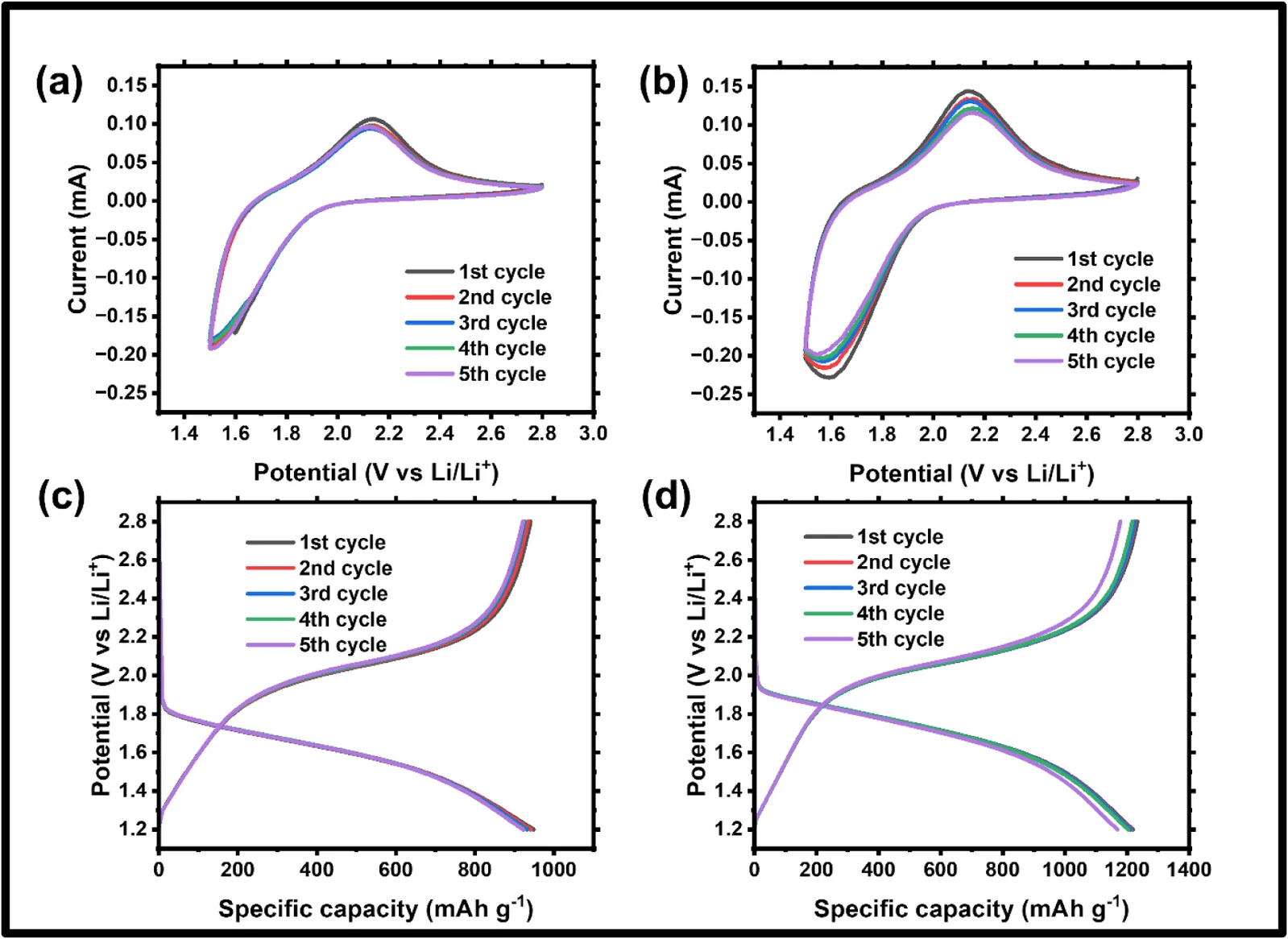
Cyclic voltammetry (CV). (**a**) 3S2UMC-ST. (**b**) 3S2UMC-DcP-based cells. Electrochemical performances of the Li/S/UMC half-cell in the voltage window of 1.2–2.8 V vs Li^+^/Li. (**c**) Cycling performance of 3S2UMC-ST based cell at 50 mA g^–1^, and (**d**) Cycling performance of 3S2UMC-DcP based cell at 50 mA g^–1^.

The influence of DcP becomes even more evident when examining the galvanostatic charge–discharge behavior at 50 mA g^–1^ (Figure 4c–d). At this relatively low current density, mass transport limitations are minimal, and the cell response is dominated primarily by intrinsic reaction kinetics and interfacial processes. The DcP-containing cell exhibits noticeably flatter discharge plateaus and reduced voltage hysteresis between charge and discharge compared to the standard electrolyte cell. This reduced hysteresis is a clear indication of lower polarization and more efficient sulfur redox conversion.

Importantly, the preservation of well-defined two-step discharge plateaus over repeated cycles in the DcP cell, compared to the gradual distortion observed in the standard electrolyte cell, suggests that the accumulation of insulating polysulfide decomposition products on the cathode surface is mitigated when DcP is present. Such a distortion of the voltage plateau is commonly associated with increasing interfacial resistance and loss of active surface area during cycling.

These observations directly correlate with the CV results shown in Figure 4a–b. The sharper redox peaks and lower polarization observed in CV are reflected in the more stable and less polarized charge–discharge profiles at 50 mA g^–1^. Together, these results provide early electrochemical evidence that DcP promotes a more favorable cathode/electrolyte interphase, improving reaction kinetics from the very beginning of cycling. Extended cycling profiles at 50 mA g^–1^ for both electrolytes are presented in Figure S1 (Supporting Information), showing the same trend over prolonged cycling.

### 3.3 Mechanistic Insight: Reduced Interfacial Resistance in the Presence of DcP

Adding DcP to the electrolyte produces complementary effects consistent with improved cathode kinetics and interfacial stabilization. Electrochemical impedance spectroscopy (EIS) on full cells shows a consistently reduced charge-transfer resistance (Rct) and a stabilized low-frequency response for cells containing DcP after formation cycling (Figure 5).

**Figure 5:**
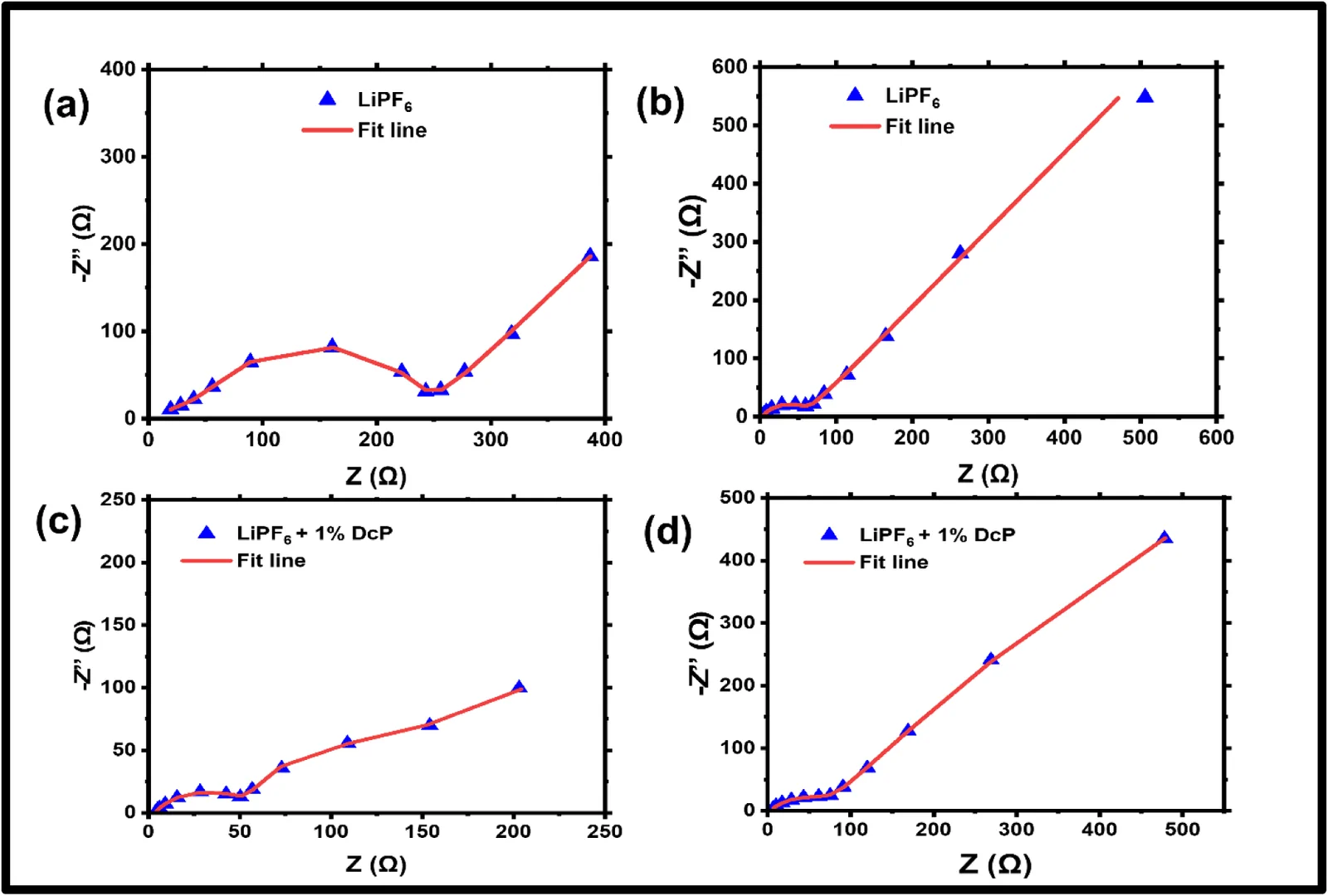
Electrochemical impedance spectroscopy. Nyquist plots and equivalent-circuit fit of a fresh Li/3S2UMC-st in the (**a**) Initial state, (**b**) same cell after long-term cycling, and a fresh Li/3S2UMC-DcP coin cell in the (**c**) Initial state. (**d**) Same cell after long-term cycling showing reduced charge-transfer resistance in cells containing DcP after formation cycling.

Figure 5 compares the Nyquist plots of the standard electrolyte cell and the DcP-containing cell, both in the initial state and after long-term cycling. In the fresh state, the semicircle in the mid to-high frequency region reflects the interfacial charge-transfer processes coupled with interphase resistance. After cycling, the standard electrolyte cell shows a clear increase in the semicircle diameter, indicating growth of interfacial resistance during operation. In contrast, the DcP-containing cell maintains a smaller semicircle and exhibits a more stable impedance response after cycling, demonstrating that the interfacial resistance increases less strongly when DcP is present. This observation directly supports the earlier electrochemical evidence, as in Figure 4a–b, where the DcP-containing cell shows sharper redox peaks and lower polarization, and in Figure 4c–d, it displays reduced voltage hysteresis during charge/discharge. The EIS results provide mechanistic support for these trends: a lower and more stable Rct implies that the cathode/electrolyte interface remains more ion-conductive and kinetically favorable during cycling, which translates into reduced polarization in both CV and galvanostatic profiles. The stabilized low-frequency region in the DcP cell (Figure 5) also suggests improved mass-transport behavior at the electrode/electrolyte interface after cycling. While UMC confinement (Figures 1–3) primarily limits polysulfide escape by physical trapping, DcP appears to further regulate the interfacial environment, reducing the buildup of resistive surface layers. This is consistent with the microstructural role of UMC: a highly confined sulfur phase can reduce polysulfide release, but the conversion reactions still occur at the interface; therefore, stabilizing that interface is necessary to preserve fast kinetics over time. Elemental mapping confirming uniform sulfur distribution before cycling is shown in Figure S2, supporting that the impedance evolution originates from interfacial changes rather than compositional inhomogeneity.

Overall, the EIS results suggest that DcP not only improves the initial electrochemical response but also helps maintain a lower interfacial resistance after cycling. This provides a clear link between structure and function: UMC confines sulfur and suppresses uncontrolled dissolution, while DcP stabilizes the cathode/electrolyte interphase and maintains charge-transfer kinetics. These combined effects are expected to lead to improved rate performance and cycling stability, which is evaluated in the following section.

### 3.4 Rate Capability and Cycling Performance under Increased Current Load

The practical consequence of the improved interfacial behavior observed in Figure 4 and the reduced charge-transfer resistance shown by EIS (Figure 5) becomes clearly visible when the cells are subjected to higher current densities. Figure 6 compares the rate capability and cycling performance of the 3S2UMC cathode in standard and DcP-modified electrolytes.

**Figure 6:**
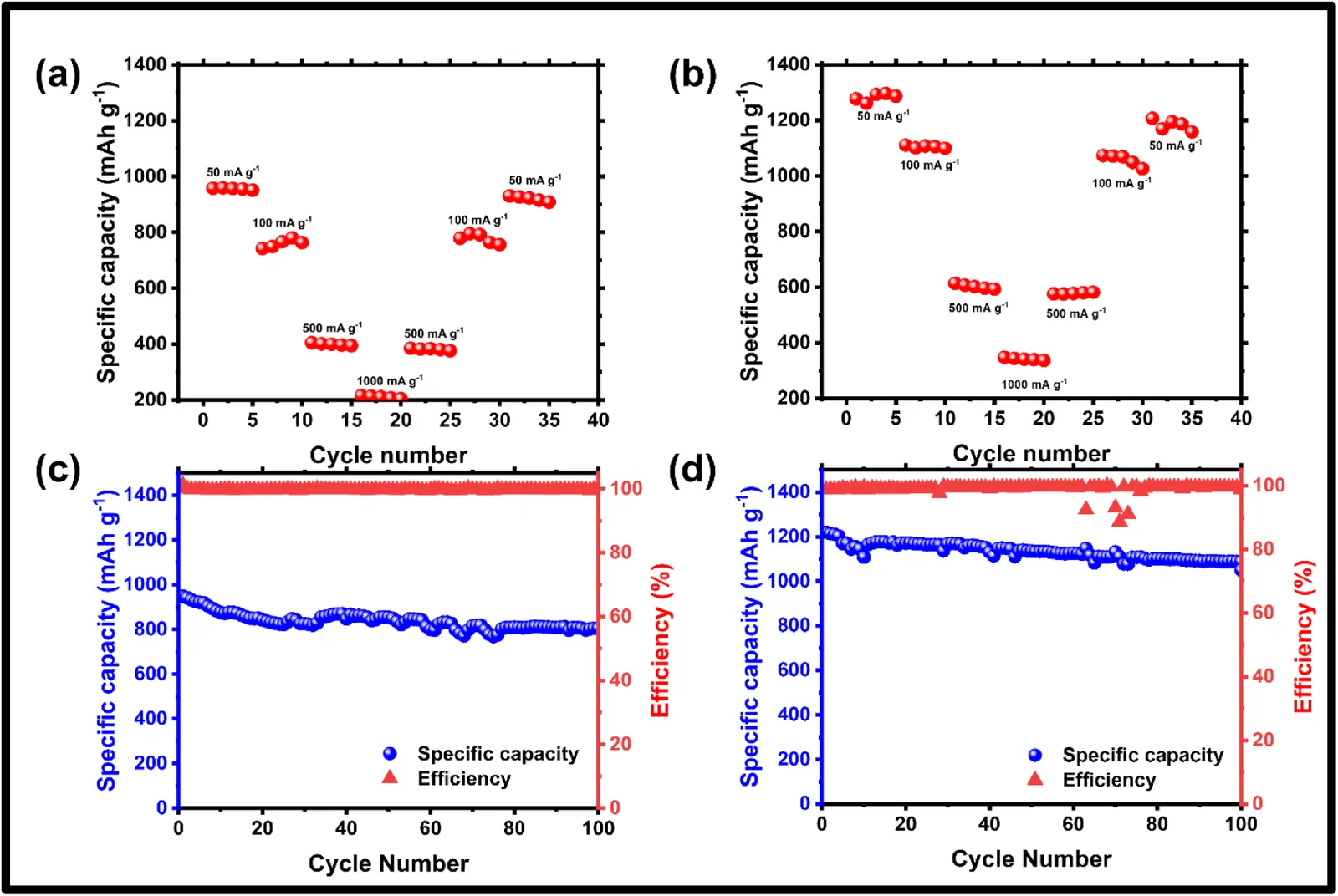
Rate capability and cycling stability reflecting improved interfacial kinetics in the presence of DcP. (a) Rate-capability performance of the 3S2UMC cathode in carbonate-based electrolyte. (b) Rate-capability performance of the 3S2UMC cathode in DcP-modified carbonate-based electrolyte. (c) Cycling performance at 100 mA g^–1^ in carbonate-based electrolyte. (d) Cycling performance at 100 mA g^–1^ in DcP-modified carbonate-based electrolyte, showing improved capacity retention under increased current load.

As shown in Figure 6a–b, the DcP-containing cell maintains higher discharge capacities across the entire range of applied current densities. The difference between the two cells becomes increasingly pronounced as the current density increases. At higher rates, where polarization and kinetic limitations typically dominate Li–S performance, the DcP cell exhibits noticeably smaller capacity loss. This directly reflects the lower interfacial resistance previously observed in the EIS measurements (Figure 5).

The rate behavior also correlates strongly with the charge–discharge characteristics observed at low current density (Figure 4c–d). The reduced voltage hysteresis and more stable discharge plateaus seen at 50 mA g^–1^ are now manifested as improved capacity retention when the electrochemical reactions are forced to proceed faster. In other words, the kinetic advantage introduced by DcP at low current density becomes even more critical under high-rate conditions.

Cycling performance at 100 mA g^–1^ (Figure 6c–d) further highlights this difference. The DcP-containing cell retains its capacity more effectively over repeated cycles, while the standard electrolyte cell shows more pronounced capacity fading. This trend is consistent with the interpretation that DcP helps to preserve a stable cathode/electrolyte interphase, preventing the progressive buildup of resistive surface layers during cycling.

Importantly, this behavior must also be viewed in the context of the UMC structure (Figures 1–3). The ultramicroporous carbon confines sulfur and limits the amount of polysulfide species that can freely dissolve into the electrolyte. However, even with good confinement, the sulfur redox reactions still occur at the cathode surface, where interfacial resistance can gradually increase. The combined effect of UMC confinement and DcP interphase stabilization, therefore, becomes evident here: UMC limits polysulfide release, while DcP ensures that the reaction interface remains ion-conductive and kinetically favorable.

In order to scientifically quantify the purported benefits of DcP additive on our battery system, a pristine cathode was made from the melt diffusion of Ketjen black and sulfur powder in a 7:3 ratio. The melt diffused 7KB:3S was then used to fabricate electrodes much in the same manner as 3S2UMC (described in the study; 8:1:1, 7Kb3S:PVF Carbon black (Super C65)).

As can be observed from Figure 7, addition of DcP not only enhances the electrochemical performance of UMC based sulfur host, but it also leads to greater performance markers in a standard carbon black-sulfur composite.

**Figure 7:**
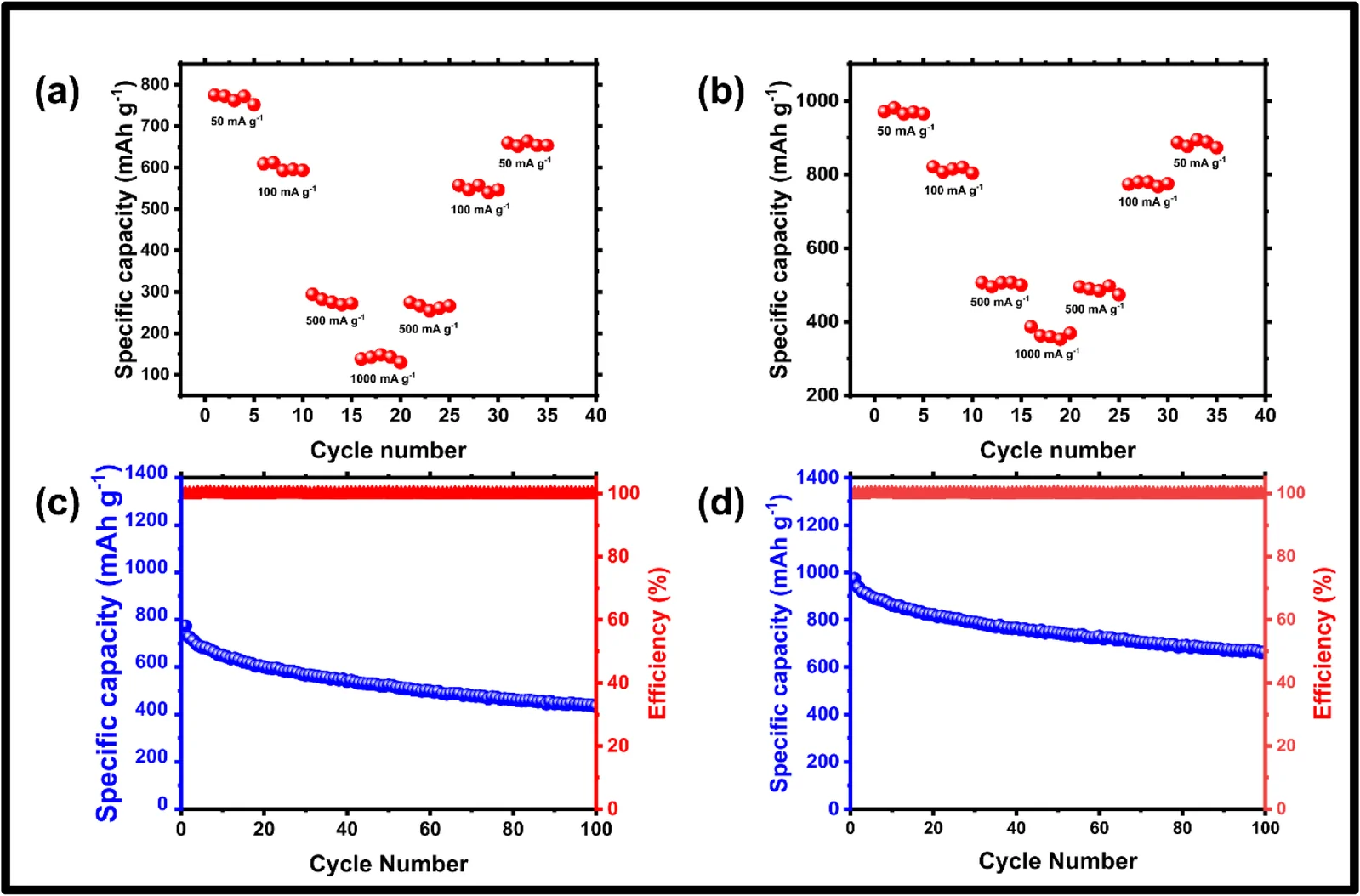
Rate capability and cycling stability reflecting improved interfacial kinetics in the presence of DcP. (a) Rate-capability performance of the 7KB3S cathode in carbonate-based electrolyte. (b) Rate-capability performance of the 7KB3S cathode in DcP-modified carbonate-based electrolyte. (c) Cycling performance at 50 mA g^–1^ in carbonate-based electrolyte. (d) Cycling performance at 50 mA g^–1^ in DcP-modified carbonate-based electrolyte.

Taken together, the results in Figure 6 suggests that the cooperative action of UMC and DcP not only improves the initial electrochemical response but also enables the cathode to operate more efficiently under demanding conditions, maintaining higher capacities and more stable cycling at elevated current densities.

The improved rate capability and cycling stability observed in Figure 6, compared to the UMC-only cell, indicate that adding DcP further modifies the interfacial kinetics. Thus, UMC confinement addresses sulfur retention (baseline performance), and the DcP additive further improves Li^+^ transport and interface stability (enhanced performance).

However, electrolyte additives in lithium-based systems often influence both the cathode and the lithium metal anode. Therefore, it is important to determine whether the beneficial effect of DcP originates from stabilization of the lithium surface, or from regulation of the cathode/electrolyte interphase where sulfur conversion reactions occur. To distinguish between these possibilities, Li//Li symmetric cells were examined under identical electrolyte conditions.

### 3.5 Li Symmetric Cell Behavior: Identifying the Primary Action Site of DcP

Voltage response during continuous lithium plating/stripping at 0.5 mA cm^–2^ with a capacity density of 0.5 mAh cm^–2^ in (a) 1 M LiPF_6_ carbonate electrolyte and (b) 1 M LiPF_6_ carbonate electrolyte containing 1% DcP. Figure 8a–b shows the evolution of the voltage profile during lithium plating and stripping in Li//Li symmetric cells at this current density. The cell using 1 M LiPF_6_ carbonate electrolyte (Figure 8a) shows stable voltage profiles over 200 h with a Coulombic efficiency consistently higher than 99% over 100 cycles. The voltage profile of the cell using 1 M LiPF_6_ + 1% DcP (Figure 8b) shows rapidly increasing polarization, leading to fluctuating and increasing overpotential after approximately 25 h (voltage spiking), indicating the accumulation of an evolving SEI and ubiquitous local short circuits.

**Figure 8:**
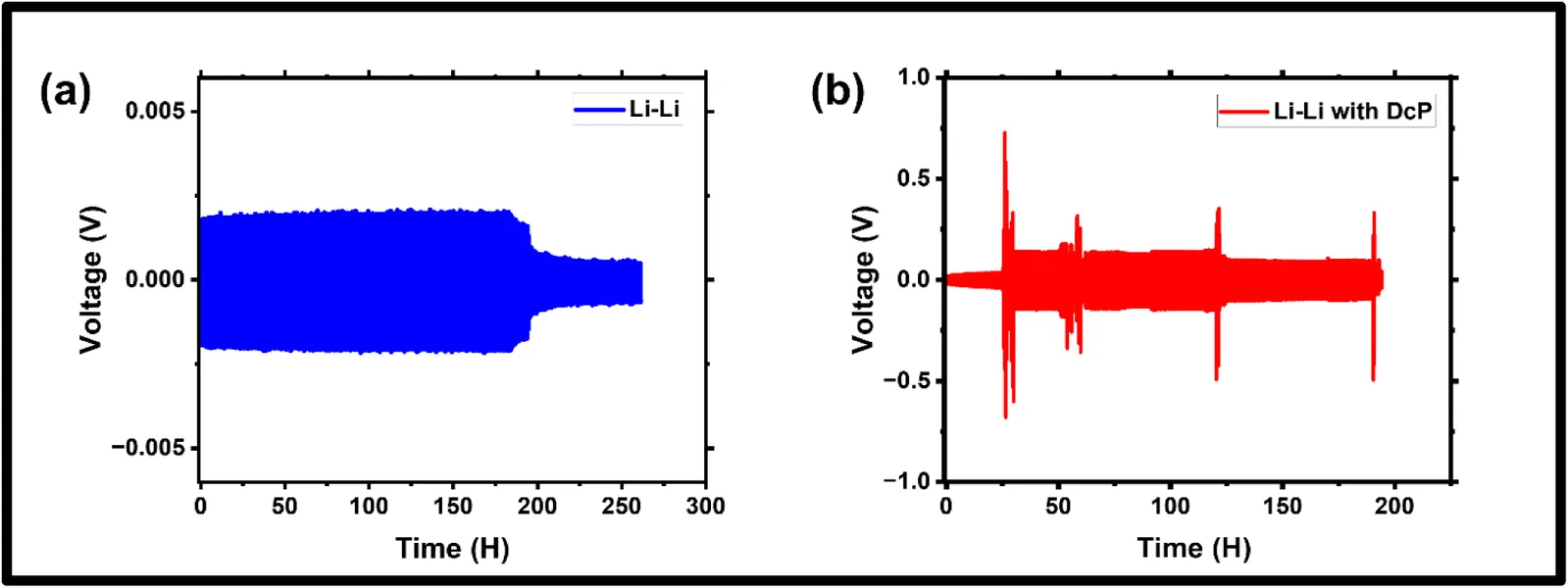
Li//Li symmetric cell voltage–time profiles revealing the primary action site of DcP.

Such behavior is often observed with carbonate-based electrolytes, where continuous Li/electrolyte reactions occur, causing severe dead Li formation and SEI accumulation that eventually block ion-conductive pathways.^41–44^ A peculiar observation is that, although the voltage–time profile of the modified electrolyte cell displays voltage spiking, the Coulombic efficiency remains greater than 99% consistently throughout the measurement.

At first glance, this behavior may appear contradictory to the improved performance observed in the Li–S cell. However, we confirmed that the stable low-voltage profile in the DcP-containing symmetric cells was consistently observed in repeated tests, indicating reproducible behavior. This comparison therefore reinforces that DcP does not primarily modify the Li-metal SEI, but instead acts at the cathode interface where Li^+^ transport and sulfur redox reactions occur. The full cells containing DcP exhibit reduced voltage hysteresis in the charge–discharge curves (Figure 4c–d), lower and more stable charge-transfer resistance (Figure 5), and improved rate capability and cycling stability (Figure 6). These improvements occur even though DcP does not improve lithium plating/stripping behavior in the symmetric cell configuration.

In contrast, when no sulfur is present (Li|Li cells), DcP produces no notable improvement (both cells become stable at low overpotential), indicating that the additive’s benefit is tied to the sulfur cathode, this comparison indicates that DcP does not primarily function as a lithium-protective additive in carbonate electrolyte. Instead, its principal beneficial action occurs at the cathode/electrolyte interface, where sulfur redox reactions take place. In the full cell, the presence of confined sulfur within the ultramicroporous UMC structure (Figures 1–3) creates a different electrochemical environment compared to bare lithium metal. Under these conditions, DcP interacts with the sulfur conversion process and stabilizes the interfacial region where Li^+^ transport and sulfur redox reactions occur.

Therefore, the symmetric cell results help to localize the action of DcP within the battery system. While DcP influences lithium deposition behavior in carbonate electrolyte, its positive contribution to Li–S performance arises from interfacial stabilization at the sulfur cathode rather than modification of lithium plating/stripping processes. Combined with the Li–S cycling data (Figures 4–6), these results indicate that DcP’s benefit is observed only in the full sulfur cells, confirming that its primary action site is the cathode interface, not the lithium anode.

Having established that DcP does not primarily influence lithium deposition behavior, the focus must return to the cathode, where the beneficial electrochemical effects were consistently observed. If DcP indeed stabilizes the cathode/electrolyte interphase during sulfur conversion, this effect should be reflected in the postcycling morphology of the electrode. Therefore, the cycled cathodes were examined using SEM and elemental mapping to directly visualize how the surface structure and sulfur distribution evolve in the presence and absence of DcP.

### 3.6 Postcycling Cathode Morphology and Interpretation

Postcycling SEM and elemental mapping of the cathodes show clear differences between cells with and without DcP. The standard electrolyte cell accumulates a thick, heterogeneous deposit layer consistent with trapped polysulfide decomposition products, whereas the 3S2UMC + DcP cathode surface is comparatively cleaner and more uniform (Figure 8e–h). A more detailed morphological comparison, including pristine cathodes and localized deposit regions after cycling with and without DcP, is provided in Figures S3–S6. These additional images reveal the extent of deposit accumulation and sulfur migration at higher magnifications, supporting the observations presented in Figure 8.

Elemental EDS maps confirm lower sulfur migration toward the separator and reduced binder-rich surface coverage in DcP cells (Figure 9). These morphological observations corroborate the interpretation that UMC confinement reduces initial polysulfide generation and that DcP further limits deposit buildup by fostering an interphase that spatially localizes reaction products and promote reversible redox processes.

**Figure 9:**
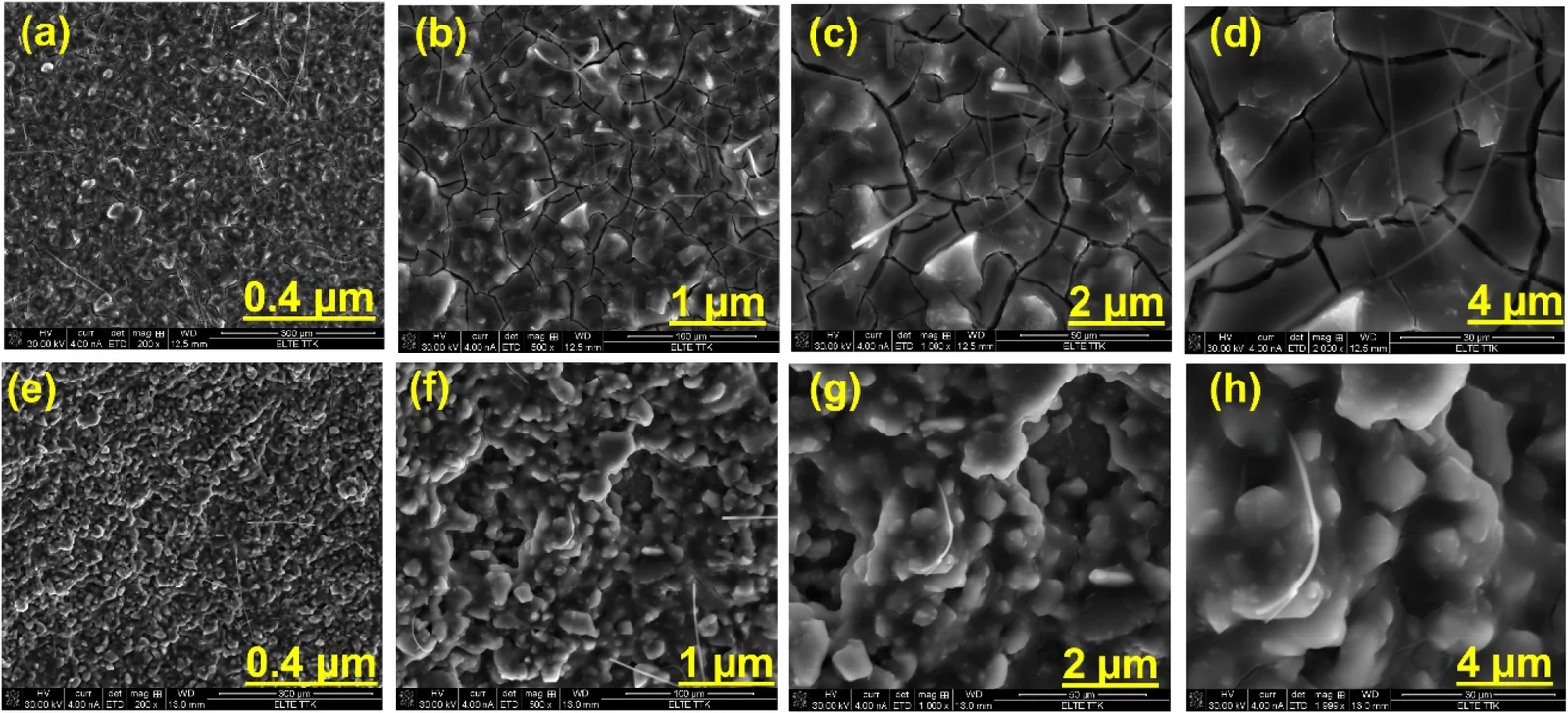
Postmortem cathode morphology after cycling. Comparative SEM images of cathodes cycled without (a–d) and with (e–h) DcP, showing reduced insulating deposits and more uniform surface in the DcP cell.

This direct visual evidence is consistent with the electrochemical trends discussed earlier. The reduced voltage hysteresis observed in the charge–discharge curves (Figure 4), the lower and more stable charge-transfer resistance revealed by EIS (Figure 5), and the improved rate and cycling performance (Figure 6) all indicate that the cathode/electrolyte interface remains more stable in the presence of DcP.

Combining the structural confinement offered by UMC with the ion-conductive interphase induced by DcP yields three cooperative effects that explain the electrochemical outcomes: (i) reduced active material loss: UMC’s ultramicropores physically limit polysulfide diffusion into the bulk electrolyte (Figure 2); (ii) enhanced local Li^+^ availability: the DcP-derived interphase lowers local Rct and improves Li^+^ transport to sulfur reaction sites (Figure 5); and (iii) preserved electrode architecture: together they prevent thick insulating deposits from forming on the cathode surface, maintaining electrical connectivity and active surface area (Figure 9).

XPS investigation of the used cathodes was conducted to compare a standard cathode with a DcP-modified cathod. Both samples contained expected cathode elements (C, O, F) and electrolyte-derived species (Li, P), alongside trace amounts of Al, Si, and Na. Analysis revealed that both cathodes developed complex solid-electrolyte interface (SEI) layers composed of similar organic and inorganic compounds, such as LiF, carbonates, and various oxidized phosphorus species (Figure 10). The most significant structural difference was observed in the C 1s region, where the standard cathode displayed a broad, unstructured band, indicating that graphitic carbon parts were covered by a thick (>10 nm) SEI layer. In contrast, graphitic carbon signals remained observable in the DcP cathode at 284.4 eV, suggesting that the DcP-modified SEI layer was either thinner or less continuous. Additionally, there were clear differences in the abundance of oxidized carbon species, with the DcP cathode exhibiting a stronger accumulation of carbonyl-containing species at 287.7 eV compared to the standard cathode.

**Figure 10:**
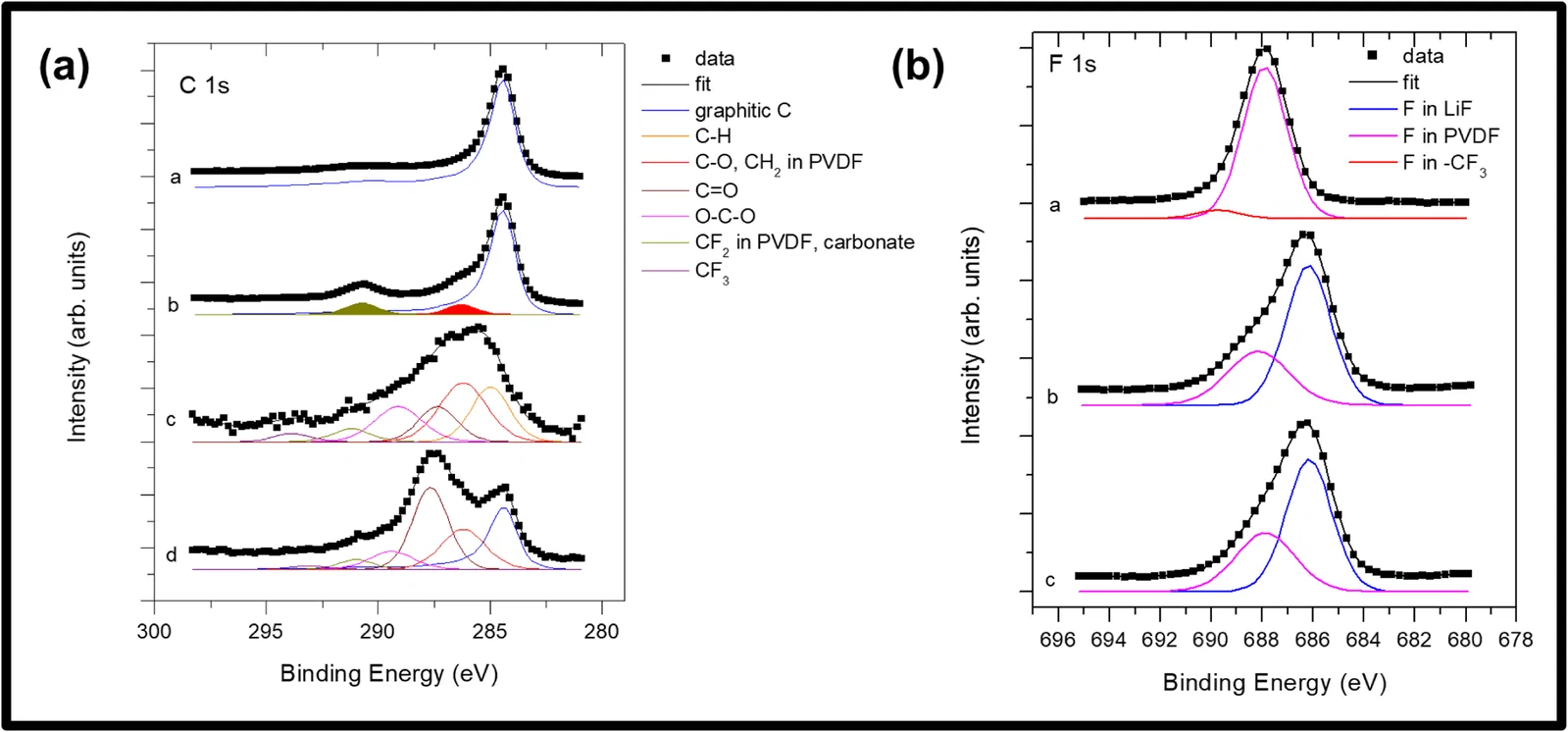
(a) C 1s spectra of a): a highly graphitic carbon black sample (Super P); b): a sulfur-C cathode prepared with the PVDF binder; c): the used standard cathode; d): the used DcP cathode. (b) F 1s spectra of a): a sulfur-C cathode prepared with PVDF binder; b): the used standard cathode; c): the used DcP cathode.

Regarding other spectral features, the S 2p peaks for both cathodes were located at 169.7–169.8 eV, indicating the presence of sulfate groups, although significant sulfur sublimation was evident. The F 1s spectra for both used cathodes were essentially identical, both dominated by a strong peak at 686.2 eV attributed to LiF. This contrasts with the initial state of a sulfur-C cathode with PVDF binder, where the F 1s peak was located at 687.9 eV, consistent with CF2 groups. Furthermore, both cathodes exhibited very similar Li 1s spectra with a single peak at 56.5 eV and P 2p spectra modeled by a single peak at 135.2 eV, both consistent with electrolyte decomposition products. In conclusion, while the surface species are qualitatively similar, the DcP modification results in a distinct SEI morphology and thickness, as reflected by the differences in the C 1s spectra.

These observations also emphasize that effective improvement of Li–S batteries require simultaneous control over both sulfur behavior inside the host structure and the interfacial chemistry at the reaction surface. While UMC limits the physical escape of sulfur species, DcP regulates the chemical environment in which these species are reversibly converted during cycling. The preserved cathode morphology, together with the stabilized electrochemical response, demonstrates that the degradation pathways typically observed in carbonate-based Li–S systems can be significantly mitigated when confinement and interphase regulation act in concert. This combined effect explains why the performance improvement is consistently observed across CV behavior, impedance evolution, rate capability, and long-term cycling, and is ultimately reflected in the postcycling structural integrity of the electrode. The Supporting Information, therefore, provides extended morphological evidence that complements Figure 9 and reinforces the interpretation of interfacial stabilization in the presence of DcP.

## 4 Conclusion

In summary, the incorporation of DODT–PEG copolymer as an electrolyte additive, in combination with a PVDF-derived ultramicroporous carbon (UMC) sulfur host, has been demonstrated to markedly enhance lithium–sulfur battery performance in carbonate-based electrolyte. The resulting 3S2UMC cathode delivers an initial discharge capacity of 1219 mAh g^–1^ and sustains a Coulombic efficiency consistently higher than 99% over 100 cycles, alongside robust rate capability across multiple C-rates.

Electrochemical analysis shows reduced voltage hysteresis in the charge–discharge profiles, while impedance measurements, and we propose that the DODT–PEG copolymer adsorbs onto the cathode, forming a polymer layer that can facilitate Li^+^ transport at the interface. At the same time, the UMC host confines small sulfur allotropes within its ultramicroporous structure, suppressing polysulfide dissolution and mitigating the effects of volume expansion. Postcycling morphology further verifies that the cathode surface remains significantly cleaner in the presence of DcP, reflecting preserved interfacial integrity.

Together, these complementary effects produce stable cycling behavior and high reversible capacity, demonstrating that performance improvement arises from the combination of sulfur confinement and interphase stabilization rather than from either component alone. This work highlights a simple and scalable strategy for improving Li–S batteries by simultaneously tailoring the sulfur host structure and the electrolyte chemistry. These findings establish a clear mechanistic framework for cooperative host–electrolyte design in carbonate-based Li–S systems.

## Supplementary Material



## Data Availability

The data that support the findings of this study are available from the corresponding author upon reasonable request.

## Notes

The ESI file contains additional electrochemical cycling data and results on structural characterisations (DOCX)

## Notes

D.O.-O.: Methodology, validation, investigation, analysis, writing – original draft; L.S.S.: Investigation, analysis, validation; P.N.: Investigation, resources, analysis; M.S.: Resources, analysis; D.U.: Instrumentation facility, analysis; Á.S.: Resources, investigation, analysis, validation; B.I.: Funding acquisition, methodology, resources, knowledge transfer, analysis, validation, review and editing; R.K.: Funding acquisition, review and editing, supervision
