# Supplementary material for: Stabilizing Lithium–Sulfur Batteries in Carbonate Electrolytes via DODT–PEG Interphase Engineering and Ultramicroporous Carbon Confinement
Source: ACS Omega. 2026 Aug 25;11(35):53278–90. doi: 10.1021/acsomega.6c06179 (PMC13563599; doi:10.1021/acsomega.6c06179)
Supplement: Supplementary file 1 [file ao6c06179_si_001.docx]

**Supporting Information**

**Stabilizing Lithium–Sulfur Batteries in Carbonate Electrolytes via DODT–PEG Interphase Engineering and Ultra-Microporous Carbon Confinement**

Derek Ovc-Okene ^a,b^, Lakshmi Shiva Shankar ^c^, Péter Nagy ^d^, Marton Szabados ^e^, Dávid Ugi ^a,g^ , Ákos Szabó ^f^*, Béla Iván ^f^*, Róbert Kun ^a,h,^*

*^a^* *Renewable Energy Research Group, Institute of Materials and Environmental Chemistry, HUN-REN Research Centre for Natural Sciences, H-1117 Budapest, Magyar tudósok krt. 2., Budapest, Hungary*

*^b^* *Department of Chemical and Environmental Process Engineering, Faculty of Chemical Technology and Biotechnology, Budapest University of Technology and Economics, Műegyetem rkp. 3, H-1111 Budapest, Hungary.*

*^c^ Zalaegerszeg Innovation Park, Széchenyi István University, Dr. Michelberger Pál út 3., H-8900 Zalaegerszeg, Hungary*

*^d^ Department of Physical Chemistry and Materials Science, University of Szeged, Rerrich Béla tér 1, Szeged, H-6720, Hungary*

*^e^ Department of Applied and Environmental Chemistry, University of Szeged, Rerrich Béla tér 1, Szeged H-6720, Hungary*

*^f^ Polymer Chemistry and Physics Research Group, Institute of Materials and Environmental Chemistry, HUN-REN Research Centre for Natural Sciences, H-1117 Budapest, Magyar tudósok krt. 2., Budapest, Hungary*

*^g^ Department of Materials Physics, Eötvös Loránd University, Pázmány Péter sétany 1/a., 1117 Budapest, Hungary*

*^h^ Sustainability Competence Centre, Széchenyi István University, H-9026 Győr, Egyetem square 1, Hungary*

* *Corresponding authors: Dr. Robert Kun (Tel: +36 1 382 6579; E-mail:* [*kun.robert@ttk.hu*](mailto:kun.robert@ttk.hu)*), Dr. Ákos Szabó (Tel : +36 1 382-6511,* [*szabo.akos@ttk.hu*](mailto:szabo.akos@ttk.hu)*), Béla Iván (Tel : +36 1 382 6512, ivan.bela@ttk.hu)*

**Figure S1.** Cycling performance of (top row) 3S2UMC-ST-based cell; and (bottom row) 3S2UMC-DcP-based cells at 50mA g^-1^.

**Figure S2.** Elemental analysis by EDS of (a): UMC, (b): 3S2UMC.

**Figure S3.** SEM images of (a-e) pristine 3S2UMC cathode under different magnification, and (f-g) EDS elemental analysis of pristine 3S2UMC cathode.

 **Figure S4.** SEM images of (a-e) 3S2UMC cathode after 100 cycles using standard electrolyte, under different magnification, and (f-g) EDS elemental analysis of 3S2UMC cathode after 100 cycles using standard electrolyte.

**Figure S5.** SEM images of (a-e) 3S2UMC cathode (localized spot deposit) after 100 cycles using standard electrolyte, under different magnification, and (f-g) EDS elemental analysis of 3S2UMC cathode (localized spot deposit) after 100 cycles using standard electrolyte.

**Figure S6.** SEM images of (a-e) 3S2UMC cathode after 100 cycles using standard electrolyte with DcP additive, under different magnification, and (f-g) EDS elemental analysis of 3S2UMC cathode after 100 cycles using standard electrolyte with DcP additive.
